# Cryptic Declines in a Widespread Australian Frog Complex

**DOI:** 10.1002/ece3.73384

**Published:** 2026-04-14

**Authors:** Jordann Crawford‐Ash, Stephanie Pulsford, Will Osborne, Danswell Starrs, Geoffrey W. Heard, Ben C. Scheele

**Affiliations:** ^1^ Fenner School of Environment and Society Australian National University Canberra Australian Capital Territory Australia; ^2^ Office of Nature Conservation City and Environment Directorate, ACT Government Canberra Australian Capital Territory Australia; ^3^ Institute for Applied Ecology University of Canberra Canberra Australian Capital Territory Australia; ^4^ ACT Office of Water City and Environment Directorate, ACT Government Canberra Australian Capital Territory Australia; ^5^ Ecology and Evolution, Research School of Biology Australian National University Canberra Australian Capital Territory Australia; ^6^ Terrestrial Ecosystem Research Network and Centre for Biodiversity and Conservation Science The University of Queensland Brisbane Queensland Australia

**Keywords:** alpine ecology, amphibian declines, *Batrachochytrium dendrobatidis*, disease ecology, occupancy modeling, population monitoring

## Abstract

Amphibian populations in upland areas have experienced disproportionately high rates of decline worldwide, yet the status of many remains poorly understood due to limited systematic surveys and long‐term monitoring. In eastern Australia, the *Pseudophryne bibronii* complex ( *P. bibronii* and *P. dendyi* ) was once widespread and common, but its current population status is uncertain. We aimed to quantify contemporary occupancy, relative abundance, and disease prevalence in this group by surveying 70 historically occupied sites spanning an elevational gradient from near sea level to ~1700 m elevation. Sites were surveyed up to six times over two breeding seasons, and adult males were swabbed to determine infection rates with the fungal pathogen *Batrachochytrium dendrobatidis* (Bd). Occupancy declined sharply with elevation, from 71% at low elevations to 28% in upland areas, with counts of calling males showing a similar pattern. Bd prevalence increased from 6.7% at low elevations to 29.2% in upland sites, with the highest infection intensities also recorded at higher elevations. These findings implicate Bd as a potential contributor to upland declines, possibly alongside other environmental pressures. Our results demonstrate that common species can undergo substantial, unrecognized contractions in the absence of targeted monitoring and provide an example of time‐lagged declines potentially associated with chytrid fungus.

## Graphical abstract

The study surveyed 70 historical sites across an elevation gradient in south‐eastern Australia to assess the status of the *Pseudophryne bibronii* complex ( *P. bibronii* and *P. dendyi* ) and Bd infection. Occupancy and abundance declined sharply with elevation (71% at low elevations versus 28% high elevations), while Bd prevalence rose (6.7% to 29.2%), suggesting Bd, possibly with other stressors, contributes to upland declines. The results show how “common” species can undergo substantial, largely unnoticed contractions without targeted long‐term monitoring.

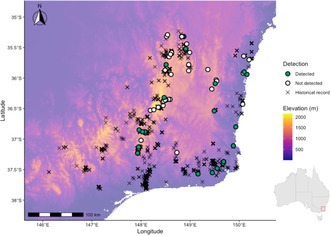

## 1 Introduction

Understanding species population trajectories is a fundamental challenge in biodiversity conservation, particularly in the context of accelerating global change. Despite widespread concern about species loss, most conservation assessments are underpinned by limited or incomplete data on population trends (Lindenmayer et al. 2012; Wintle et al. 2019; Oliver et al. 2021). Even among species formally listed as threatened, many lack systematic, long‐term monitoring, undermining assessments of extinction risk and prioritization of conservation actions (Proença et al. 2017; Scheele, Legge, et al. 2019). For example, in Australia, population trends are not monitored for approximately one‐third of listed threatened vertebrates, and many that are monitored are not tracked over meaningful time scales (Legge et al. 2018; Scheele, Legge, et al. 2019). As a result, declines may go unnoticed, particularly in taxa with patchy distributions, cryptic behavior, or complex life histories (Donaldson et al. 2016; Scheele, Legge, et al. 2019).

The rapid decline of many amphibian species over the past four decades illustrates the critical value of systematic surveys and monitoring. Since the early 1980s, amphibians have experienced the most rapid declines of any vertebrate group, with 42% of species now listed as threatened under the IUCN Red List (Luedtke et al. 2023). These declines are driven by multiple threats including disease, habitat loss, invasive species, and climate change (Berger et al. 1998; Skerratt et al. 2007; Hof et al. 2011; Scheele, Pasmans, et al. 2019; Grant et al. 2020; Luedtke et al. 2023; Crawford‐Ash, Erens, et al. 2025; Crawford‐Ash, Evans, et al. 2025). Species for which long‐term monitoring exists show varied long‐term trajectories: some continue to decline rapidly (Murray et al. 2011; Scheele, Pasmans, et al. 2019; Geyle et al. 2021), while others appear to have stabilized or recovered (Retallick et al. 2004; Newell et al. 2013; Crawford‐Ash, Erens, et al. 2025), even in modified environments (Callaghan et al. 2021). However, apparent stabilization can belie the fact that threatening processes can have long‐term, less obvious effects such as suppressing abundance, constraining distributions, and reducing resilience to other environmental stressors (Murray et al. 2009; Phillott et al. 2013; Heard et al. 2015, 2024; Belasen et al. 2022; Scheele et al. 2023). Moreover, 23% of amphibians on the IUCN Red List remain Data Deficient (Luedtke et al. 2023), and many more lack consistent, long‐term monitoring.

Australia is a global hotspot for amphibian declines, with 49 species listed as threatened nationally and eight considered at high risk of extinction (Gillespie et al. 2020; Geyle et al. 2021). While trajectories of some Australian species are well documented, many remain poorly understood due to a lack of systematic survey or monitoring. Two such species are Bibron's toadlet (*Pseudophyrne bibronii*) and Dendy's toadlet ( *P. dendyi* )—small terrestrial frogs distributed collectively across much of south‐eastern Australia (Anstis 2017). Both species were historically widespread and considered common within their ranges, but local extinctions of upland populations have been suspected in recent decades (Osborne 1990; Gillespie et al. 1995; Hunter et al. 2018). These species are closely related and share similar ecological traits and may represent a species complex (Anstis 2017). Because they are difficult to differentiate by call or visual inspection, and genetic boundaries remain unresolved, we refer to them collectively as the *P. bibronii* complex here.

The *P. bibronii* complex may be particularly vulnerable to decline. Their traits mirror those of other *Pseudophryne* species that have declined severely, such as the well‐known corroboree frogs— *P. corroboree* and *P. pengilleyi—*which are both highly susceptible to Bd (Kosch et al. 2019) and rely on breeding sites with a narrow band of hydrological and thermal characteristics (Berger et al. 1998; Osborne et al. 1999; Hunter et al. 2010). The *P. bibronii* complex occurs across a broader elevational gradient than *P. corroboree* and *P. pengilleyi;* however, upland populations of the *P. bibronii* complex may be similarly at risk of decline due to colder temperatures that are associated with increased Bd impact (Woodhams and Alford 2005; Scheele et al. 2023) and climate‐driven changes to the hydroperiod of ponds and seeps that are vital for reproduction (Scheele et al. 2012, 2016; Driscoll et al. 2026).

In this study, we quantified contemporary occupancy and abundance for the *P. bibronii* complex through resurveys of historically occupied sites across a broad elevational gradient in south‐eastern Australia. We also quantified Bd prevalence and infection intensity across the gradient, and the degree of sympatry with the common eastern froglet ( *Crinia signifera* ); a known Bd reservoir host linked to declines of other upland frogs, including *P. corroboree* and *P. pengilleyi* (Hunter et al. 2010; Scheele et al. 2017; Brannelly et al. 2018). We hypothesized that *P. bibronii* complex occupancy and abundance would decline with increasing elevation, consistent with patterns in other eastern Australian frogs (Hero et al. 2005; Hunter et al. 2018). We also predicted Bd prevalence and intensity would increase with elevation, where cooler, more moist conditions favour pathogen persistence (Woodhams et al. 2003; Scheele et al. 2023). Lastly, we expected *C. signifera* abundance to negatively affect *P. bibronii* complex persistence and abundance, especially at higher elevations (Scheele et al. 2017).

## 2 Methods

### 2.1 Study Area

**FIGURE 1:**
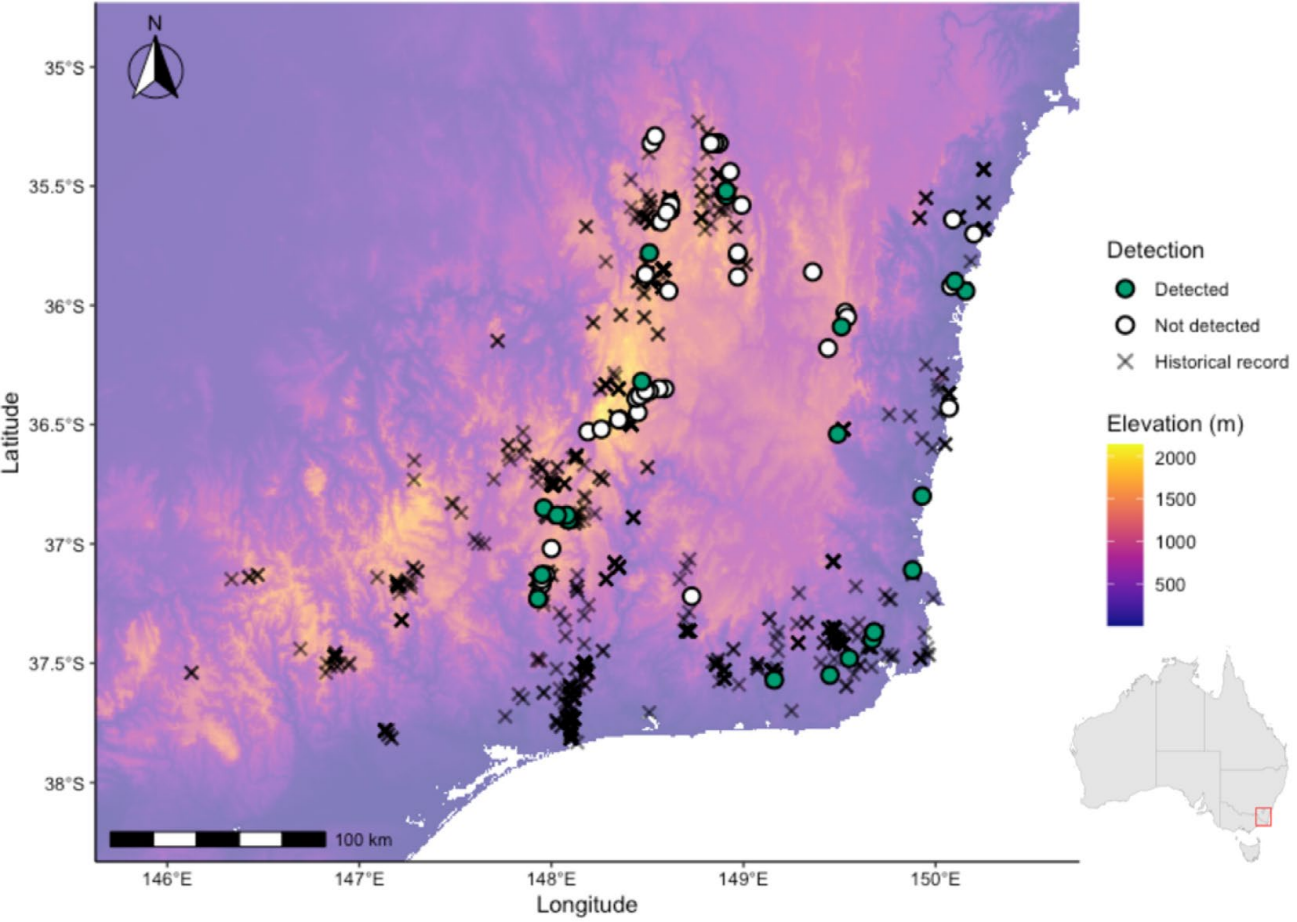
Study area and survey outcomes for *Pseudophryne bibronii* complex frogs across the Australian Capital Territory, Southern Tablelands of New South Wales and north‐eastern Victoria, Australia. Background colors show elevation (0–2000 m asl). Points denote sites surveyed in this study (green filled = detected; white filled = not detected). Gray crosses are historical species occurrence records (pre‐1990).

We compiled over 1000 historical occurrence records of the *P. bibronii* complex across south‐eastern Australia from museum collections and survey databases, restricting records to those collected prior to 1990 and located within the Australian Alps (AUA), South East Coastal Plain (SCP), and South East Corner (SEC) bioregions (Atlas of Living Australia 2025; Department of Climate Change, Energy, the Environment and Water (DCCEEW) 2025) (Figure 1). Sites were selected for field surveys based on continued site integrity (i.e., not destroyed by urbanization), accessibility, and geographic coverage. Many historical records had high spatial uncertainty. We prioritized sites with high spatial precision (≤ 1 km) but included sites with coarser data (up to 5 km uncertainty) where records were linked to locality names or clearly defined areas of suitable breeding habitat. For records with precise coordinates, surveys were centered within 1 km of the original point and targeted suitable *Pseudophryne* breeding habitat (bogs, seepage lines and ephemeral pools). For less precise records, we searched within a 5 km radius to identify appropriate breeding habitat before establishing the site (Figure 1).

Male frogs in the *P. bibronii* complex have high nest site fidelity (Byrne and Silla 2023), however, survey sites were spaced at least 1 km apart to reduce the likelihood of surveying the same individuals across locations. In total, 70 sites were surveyed across the Australian Capital Territory (ACT), New South Wales (NSW), and Victoria (VIC) (Figure 1), spanning 46 high‐elevation sites (1000–1700 m asl), 10 mid‐elevation sites (500–999 m asl), and 14 low‐elevation sites (10–499 m asl).

### 2.2 Field Surveys

We conducted audio‐visual surveys across two consecutive breeding seasons (February–April in both 2023 and 2024). Each site was surveyed up to three times per year, for six visits total. Surveys were spaced 7–10 days apart and conducted under varying weather conditions and times of day (including both day and night) to maximize detection probability based on known activity patterns (Byrne and Silla 2023).

At each visit, we surveyed all suitable breeding habitat within a 50 m radius for 15 min using call playback from the FrogID app (Rowley et al. 2019) broadcast at ~5‐min intervals. The observer recorded detection (calling or not calling) and estimated the number of calling males using ordinal categories (0, 1 = 1–5, 2 = 6–20, 3 = 21–50, 4 = 51–100 individuals). Surveys were led by the authors (JCA, WO, DS, SP), with one or two volunteers each survey assisting with call detection.

Weather data (air temperature, wind speed and relative humidity) were recorded during each survey with a Kestrel 3000 weather meter (Kestrel Instruments, Boothwyn, Pennsylvania, USA), and cloud cover was estimated visually. Additional climate variables were extracted from the NASA POWER database on a site‐by‐survey basis (Sparks et al. 2024): daily precipitation, weekly accumulated precipitation, next‐day precipitation, and average daily temperature.

### 2.3 Site‐Level Habitat Attributes

We recorded abiotic and biotic variables hypothesized to influence site occupancy for frogs in the *P. bibronii* complex, including suspected decline drivers (Table 1). Elevation was extracted manually from Google Earth Pro using precise GPS coordinates taken at the time of survey. Fire severity data from the 2019/2020 ‘Black Summer’ bushfires were obtained from the Australian Google Earth Engine Burnt Area Map (AUS GEEBAM) Fire Severity Dataset (Roff and Aravena 2020). Average canopy cover (%), site moisture (0–4 scale), and habitat disturbance (binary) were assessed visually during surveys and averaged across the six survey visits for each site. Sites lacking trees were assigned 0% canopy cover. Habitat disturbance was noted when sites showed signs of soil and vegetation disturbance from introduced mammals, human activity, or other obvious modifications. We also recorded categorical count estimates for *C. signifera* at each site at each survey (0, 1 = 1–5, 2 = 6–20, 3 = 21–50, 4 = 51–100 individuals).

**TABLE 1:** Covariates used in models of the probability of site occupancy (ψ) and detection during surveys (*p*) for the *Pseudophryne bibronii* complex. Covariates were selected based on their potential to influence occurrence and detectability, as described by ‘Ecological relevance’. Variables were measured in the field or obtained from spatial datasets (AUS GEEBAM) and climate datasets (NASA POWER).

Covariate	Model component	Description	Ecological relevance
Elevation	Occupancy	Elevation of the survey site (m), extracted from GPS coordinates	Climatic conditions, hydrology, and disease prevalence often vary along elevational gradients, affecting persistence
Fire severity	Occupancy	Fire severity from AUS GEEBAM raster	Severe burns could cause mortality, changes to vegetation, microclimate, and breeding habitat quality
Canopy cover	Occupancy	Average tree canopy cover (%) across surveys, visually estimated	Influences temperature regulation, and moisture retention
Maximum count category of *C. signifera*	Occupancy	Highest count category observed (0, 1 = 1–5, 2 = 6–20, 3 = 21–50, 4 = 51–100)	Potential reservoir for Bd; may influence occupancy via increased disease transmission
Time of survey (hour)	Detection	Hour of day survey was conducted (0–23)	Captures diel variation in calling activity
Daily temperature	Detection	Temperature at time of survey (°C)	Affects calling behavior and detectability
Next‐day precipitation	Detection	Total precipitation (mm) on the day after the survey	Linked to calling activity and breeding site conditions
Wind	Detection	Wind speed at the time of survey (m/s)	High wind may reduce calling behavior or interfere with detection
Humidity	Detection	Relative humidity (%) during survey	Affects calling behavior and frog activity
Moisture level	Detection	Categorical moisture rating from dry‐ high (0–4)	Reflects site wetness at survey time
Cloud cover	Detection	Estimated cloud cover (%) during survey	May affect calling
Habitat disturbance	Detection	Binary index (1 = disturbed, 0 = undisturbed)	May affect calling or habitat quality

### 2.4 Data Processing

All data processing and analyses were conducted in *R* version 4.4.1 (R Core Team 2024). Missing wind speed and humidity values for five ACT sites were imputed using the regional mean. Continuous covariates were z‐transformed (mean‐centered, divided by two SD) to aid model convergence and interpretation (Gelman and Hill 2007). Binary and categorical variables were not standardized.

### 2.5 Occupancy Modeling

We used single‐season occupancy models implemented with the *unmarked* R package (version 1.5.0; Fiske and Chandler 2011) to estimate site occupancy (ψ) and detection probability (*p*) for the *P. bibronii* complex and to evaluate covariate effects (Table 1). A single‐season framework was used for this study as we did not observe changes in site status between years, meeting the closure assumption (MacKenzie et al. 2002). Occupancy probability was modeled as a function of elevation, fire severity, canopy cover, and the site‐level maximum ordinal category of *C. signifera* counts (0, 1 = 1–5, 2 = 6–20, 3 = 21–50, 4 = 51–100 individuals). Detection probability was modeled with nine survey‐level covariates: daily temperature, next‐day precipitation, wind speed, humidity, soil moisture, cloud cover, habitat disturbance, and a cosine transformation of survey time (for diel patterns; see equation below). Next‐day precipitation was selected over same‐day and weekly precipitation measures to reduce collinearity and because weather conditions preceding rainfall may influence calling behavior and detection in this species. It also showed greater support in initial model fitting.

To model diel patterns in detection, we used a two‐parameter cosine function:

(1) $$\;\log\left(\frac{p_{i}}{1-p_{i}}\right)=\alpha +\beta_{1}\times \cos\left(\frac{2\pi \times {\text{hour}}_{i}}{24}\right)+\beta_{2}\times \sin\left(\frac{2\pi \times {\text{hour}}_{i}}{24}\right),$$

where *α* is the intercept, and *β*₁ and *β*_2_ are coefficients capturing diel variation. With detection covariates fixed, we fitted 15 combinations of the four site covariates (elevation, fire severity, canopy cover, *C. signifera* maximum count category) and ranked models by AICc. Final coefficients were obtained by model‐averaging across models with ΔAICc < 2 (*MuMIn* package version 1.48.4; Bartoń 2025).

### 2.6 Count Modeling

We analysed variation in *P. bibronii* complex counts (representing abundance) using cumulative‐link mixed models (CLMM; *ordinal* package version 2023.12–4.1; Christensen 2019) with a logit link. Counts were binned into ordinal categories from 1 to 4 (1 = 1–5, 2 = 6–20, 3 = 21–50, 4 = 51–100 individuals), with surveys with zero detections excluded. Site ID was included as a random intercept to account for repeated measures.

We constructed 16 candidate models based on covariates aligned with the occupancy analysis: elevation, fire severity, diel activity (night vs. day), next‐day precipitation and the maximum count category for *C. signifera* . To test whether there was a stronger reservoir host effect of *C. signifera* at higher elevations, we included an elevation × *Crinia* interaction in the candidate model set. Models were ranked by AICc, and coefficients were model‐averaged across the ΔAICc < 2 subset.

### 2.7 Disease Sampling

We swabbed 45 adult males from the *P. bibronii* complex across 13 sites spanning low, mid, and high elevations. Swabbing followed the protocols of Boyle et al. (2004), with 30 strokes across the ventral body, groin, and hind feet. Frogs were weighed and measured (SVL) for body condition. Swabs were analyzed via qPCR at CESAR laboratory (Melbourne) according to the protocols of Boyle et al. (2004) to detect Bd and quantify infection load (as ‘zoospore equivalents’).

## 3 Results

We re‐surveyed 70 sites with historical *P. bibronii* complex occurrences across south‐eastern Australia, stratified by elevation (Figure 1). Elevation was treated as a continuous covariate in all models (z‐transformed), but we report elevational bands here for descriptive summaries of detections and Bd sampling across the gradient. Frog occurrence varied markedly across elevation bands, with *P. bibronii* complex frogs found at 71.4% of low‐elevation sites (10–499 m asl), 10% of mid‐elevation sites (500–999 m asl), and 28.3% of high‐elevation sites (1000–1700 m asl) (Table S1).

Counts of *P. bibronii* complex were grouped into four ordinal categories (1 = 1–5, 2 = 6–20, 3 = 21–50, 4 = 51–100 individuals). At occupied high‐elevation sites, the lowest count category (1–5 individuals) dominated, whereas the highest category (51–100) occurred only at low‐elevation sites. The 21–50 category was likewise most frequent at low‐elevation sites (Table S2).

### 3.1 Occupancy Modeling

**FIGURE 2:**
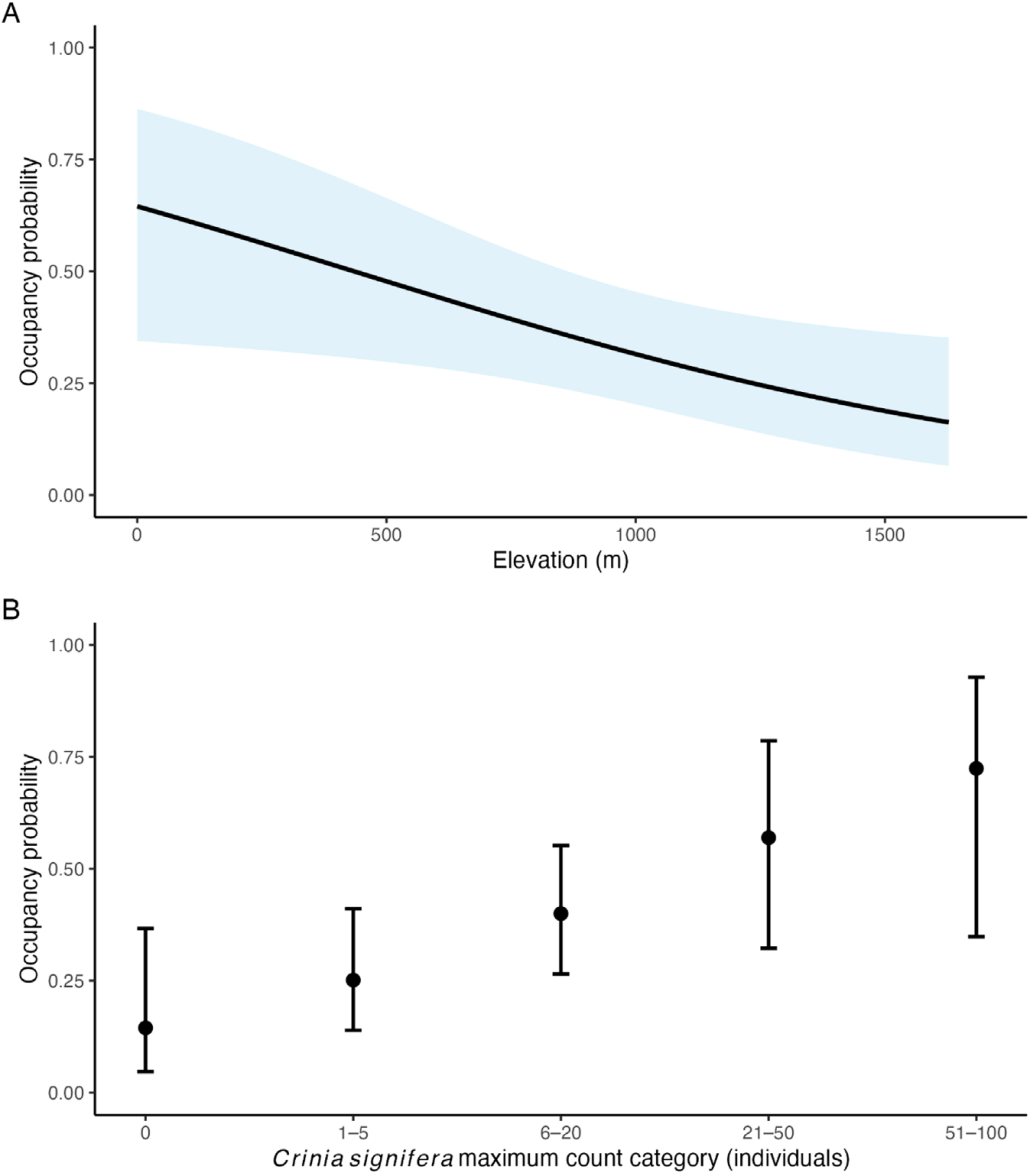
Model‐averaged relationships between predicted occupancy probability of the *Pseudophryne bibronii* complex and two site‐level covariates: (A) elevation and (B) *Crinia signifera* maximum count category (0, 1–5, 6–20, 21–50, 51–100 individuals). Shaded areas represent 95% confidence intervals.

Of the 15 candidate models, two received substantial support (ΔAICc < 2; Table S3) and were model‐averaged (Table S4). Elevation had a negative effect on site occupancy (*β* = −1.42 ± 0.62 SE, *p*‐value = 0.02; 95% CI: −2.63 – −0.20; Table S4), with the probability of occupancy declining with increasing elevation (Figure 2). In contrast, the site‐level maximum count of *Crinia signifera* was positively associated with site occupancy of *P. bibronii* complex (*β* = 1.47 ± 0.64, *p*‐value = 0.02; 95% CI: 0.22–2.72; Table S4). Fire severity showed a weak positive effect (*β* = 0.31 ± 0.54, *p*‐value = 0.57; 95% CI: 0.33–2.01) and was retained in one of the two top models but was not significant.

**FIGURE 3:**
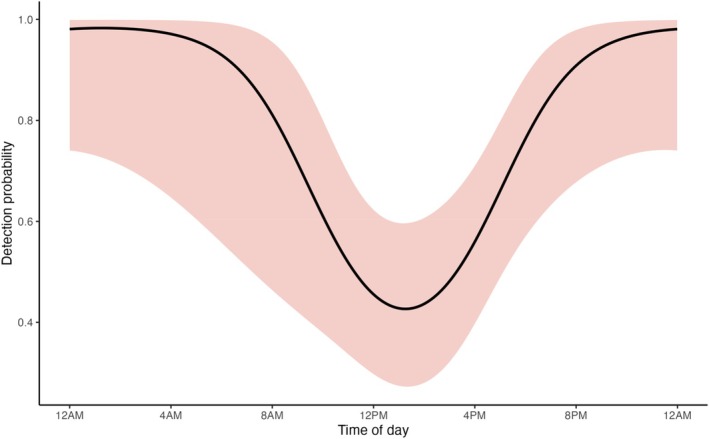
Effect of time of day on detection probability for the *Pseudophryne bibronii* complex from the top‐ranked occupancy model. The solid line is the model‐estimated mean and the shaded ribbon is the 95% confidence interval.

Detection probability was best explained by diel activity patterns, with a significant cosine‐transformed hour effect (*β* = 2.02 ± 0.78, *p*‐value = 0.01; 95% CI: 0.50–3.55; Table S5). The probability of detection peaked after nightfall, declined through the morning, reached a trough around midday, and increased into the evening (Figure 3). Other detection covariates had weak, non‐significant effects including temperature, next‐day precipitation, humidity, wind, cloud cover, soil moisture, and habitat disturbance (Table S5).

### 3.2 Count Modeling

Of the 16 candidate models, two received substantial support (ΔAICc < 2; Table S6) and were model‐averaged (Table S7). Site elevation and survey timing (night versus day) were the strongest predictors of counts of *P. bibronii* complex (Table S7). *Pseudophryne bibronii* complex counts declined with elevation (*β* = −1.52 ± 0.58 SE, *p*‐value = 0.01; 95% CI: −2.65 – −0.39; Table S7). Surveys conducted at night were also associated with higher counts (*β* = 1.33 ± 0.58, *p*‐value = 0.02; 95% CI: 0.20–2.47), consistent with higher detection probabilities after dark (as above). Next‐day precipitation and fire severity were retained in the top two models but were not significant predictors of counts. No other predictors were included in the top models, and interaction terms did not improve model performance.

**FIGURE 4:**
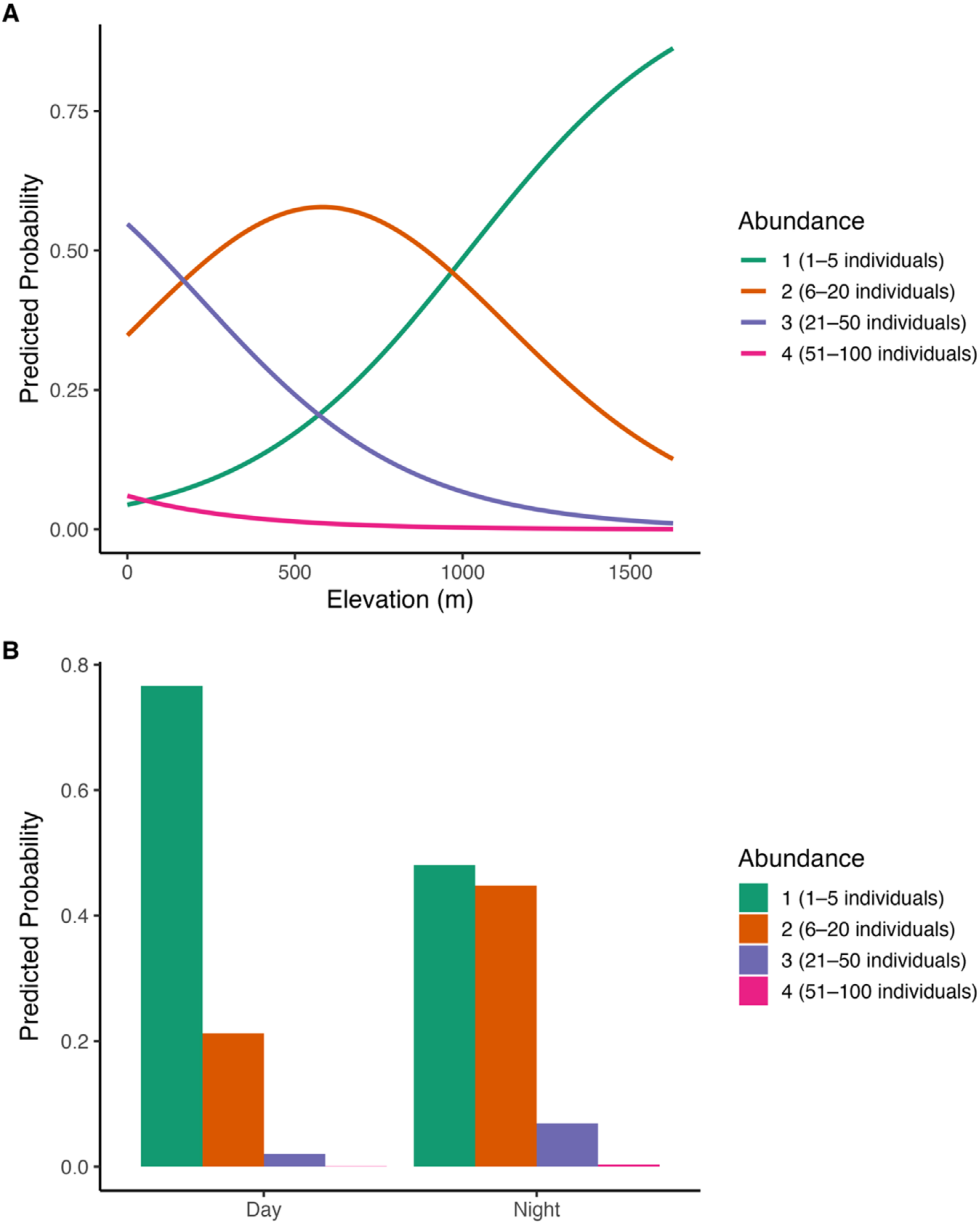
Predicted probability of each count category for the *Pseudophryne bibronii* complex from the averaged cumulative link mixed models (CLMM; models within ΔAICc < 2). Count categories are: 1–5, 6–20, 21–50, 51–100 individuals. (A) Predictions across elevation, holding other predictors at their mean or reference values. (B) Predictions by diel period (day vs. night), holding other predictors constant.

Predicted probabilities from the top‐ranked models indicated that high and mid‐elevation sites had the highest likelihood of recording the smallest count category (1–5 individuals), while high counts (≥ 21 individuals) were most probable at low elevations (Figure 4).

### 3.3 *Batrachochytrium dendrobatidis* Sampling

We swabbed 45 adult males from 13 sites: low elevation (*n* = 3 sites, 15 individuals), mid elevation (*n* = 2 sites, 6 individuals), high elevation (*n* = 8 sites, 24 individuals). Overall Bd prevalence was 18% (8 of 45 individuals positive), increasing with elevation from 6.7% at low elevation sites to 16.7% at mid elevations and 29.2% at high elevations (Table 2). The single low‐elevation positive had a low load (8.17 zoospore equivalents), whereas seven high‐elevation positives averaged 164.58 zoospore equivalents. The highest load came from the sole mid‐elevation positive (906.33 zoospore equivalents).

**TABLE 2:** *Batrachochytrium dendrobatidis* (Bd) infection rates in *Pseudophryne bibronii* complex populations across three elevation bands.

Elevation category	Total sites	Sample size	Positive swabs	Bd prevalence (% infected)
Low (10–499 m asl)	3	15	1	6.7
Mid (500–999 m asl)	2	6	1	16.7
High (1000–1700 m asl)	8	24	7	29.2

## 4 Discussion

### 4.1 Overlooked Declines in a Seemingly Secure Species

Our results reveal an elevation‐linked contraction of the *P. bibronii* complex in south‐eastern Australia. We detected frogs at 34.3% of 70 historical occurrence sites; 71.4% of lowland sites, but only 28.3% of upland sites and 10% of mid‐elevation sites. This elevational pattern was confirmed by the occupancy modeling, which accounts for imperfect detection. Abundance also declined with elevation, with generally low counts (1–5 calling males) at the few occupied upland sites, compared to higher counts (21–50 individuals) at the majority of lowland sites. Our results provide empirical evidence supporting reports of an elevation‐linked decline in the *P. bibronii* complex (Osborne 1986; Howard et al. 2010; Anstis 2017; Collins 2022; Byrne and Silla 2023). More broadly, our results contribute to a body of research showing that higher‐elevation amphibians are among the most extinction‐prone vertebrates globally (Guirguis et al. 2023). In Australia, all seven potentially extinct taxa are upland species, while the most threatened extant species, such as the southern and northern corroboree frogs ( *P. corroboree* , *P. pengilleyi* ), spotted tree frog ( *Litoria spenceri* ), Baw Baw frog ( *Philoria frosti* ), and Kroombit tinker frog ( *Taudactylus pleione* ), are also upland species (Hunter et al. 2018; Scheele, Pasmans, et al. 2019; Geyle et al. 2021).

### 4.2 Potential Drivers of Decline

While direct evidence is lacking, we suggest that the decline we document is consistent with a potential role of Bd. Although Bd sampling was limited, prevalence was highest at upland sites (29.2%) and lowest at lowland sites (6.7%), consistent with the pattern of decline severity. We also observed a high infection load in the one positive mid‐elevation sample (906.33 zoospore equivalents). With small sample sizes (*n* = 45), our Bd results should be interpreted cautiously. However, our proposition that Bd could be an important driver of the declines we document is supported by recent research that provides the first direct evidence for reduced survival in Bd‐infected *P. bibronii* in the the wild (Driscoll et al. 2026). Several mechanisms could underlie increased Bd impacts at higher elevations, leading to higher rates of local extinction. First, cooler upland temperatures favor Bd (Piotrowski et al. 2004; Sopniewski et al. 2022; Scheele et al. 2023), and second, longer frog maturation times at higher elevations reduce population capacity to persist despite increased mortality (Scheele et al. 2024). For example, age at maturation in corroboree frogs ( *P. corroboree* and *P. pengilleyi* ) increases with elevation, rising from 1 to 4 years across a gradient of 950–1600 m asl, which reduces population resilience to high adult mortality and increases the likelihood of local extinction (Scheele et al. 2024). Given the substantial elevation range of our sites (10 to ~1700 m asl), we suggest that slower maturation at high elevations, combined with higher Bd prevalence, are key mechanisms underpinning the elevational pattern of decline documented here.

Reservoir hosts can amplify Bd prevalence in sympatric species (Brannelly et al. 2018; Hudson et al. 2019; Burns et al. 2021; Wilber et al. 2022). In *P. pengilleyi* , *C. signifera* presence has been linked to increased Bd impact (Scheele et al. 2017; Brannelly et al. 2018). We predicted that counts of *C. signifera* would be negatively related to occupancy probabilities for frogs in the *P. bibronii* complex, with the possibility of an interactive effect with elevation, because the *P. bibronii* complex is likely to be less resilient to Bd at higher elevations (as above). Additionally, *C. signifera* typically has lower Bd infection prevalence at lowland sites (~10%) but very high prevalence at upland sites (Scheele et al. 2016; Brannelly et al. 2018; Crawford‐Ash and Rowley 2021). Contrary to this expectation, occupancy probability for the *P. bibronii* complex showed a positive association with counts of *C. signifera* . We interpret this relationship as likely reflecting shared environmental preferences, particularly at low elevation sites, where both species are more common, rather than facilitation at higher elevations where Bd risk is greater. Models provided no support for an interaction between elevation and *C. signifera* counts as a predictor of *P. bibronii* complex occupancy (ΔAICc > 2; Table S6). Furthermore, *C. signifera* counts showed no clear association with elevation (Spearman *ρ* = −0.12, 95% bootstrap CI −0.35 to 0.12; *p* = 0.33; Table S8; Figure S1). Accordingly, our data do not indicate suppression of *P. bibronii* and *P. dendyi* by *C. signifera* at the scales analyzed here; however, context‐specific effects particularly in high‐elevation habitats cannot be ruled out. One potential reason for the differences between our results and those documented for *P. pengilleyi* is that *C. signifera* were largely ubiquitous across our sites (detected at 62 of 70 sites), whereas in Scheele et al. (2017), some *P. pengilleyi* sites lacked *C. signifera*, and those sites had significantly lower Bd prevalence.

Unlike some eastern Australian frogs that collapsed abruptly with the emergence of Bd in the 1980s (Berger et al. 1998, 1999; Osborne et al. 1999; Hero and Morrison 2004), declines in the *P. bibronii* complex appear more gradual. Reports from the 1990s documented loss of *P. bibronii* from many former Canberra region sites (Osborne 1990; Rauhala 1997) and found few records in Victoria, mostly restricted to lower elevations in the Alps (Gillespie et al. 1995). *Pseudophryne dendyi* were historically documented in higher elevation sites (~800–1000 m) but considered insufficiently known (Rauhala 1997). Since then, there has been little systematic survey effort and a lack of long‐term monitoring, and the rate of decline has remained uncertain. Our re‐surveys of historical sites indicate that declines have occurred, but that the species still persists across much of its historical range in our study area (34.3% across the 70 surveyed sites), even in the upland regions in which it appears most exposed to Bd impacts. One explanation for the slower pace and patchier nature of declines experienced by the *P. bibronii* complex compared with other susceptible species is ecological characteristics that restrict Bd transmission. Adults of both *P. bibronii* and *P. dendyi* are highly terrestrial and lay eggs in terrestrial nests that are washed into nearby water bodies with flooding rains, sometimes weeks after breeding (Gillespie et al. 1995). Therefore, the adults are likely to have reduced contact with aquatic habitats and motile zoospores of Bd, reducing rates of contagion and infection prevalence (Kriger et al. 2007; Ruggeri et al. 2018). It is notable that infection prevalence among the breeding males swabbed during this study was low, at 18% overall.

Another factor that could have contributed to the decline of the *P. bibronii* complex is recruitment failure during drought. While droughts are not an unusual occurrence in the study region, their severity and frequency are increasing due to rising temperatures and long‐term reductions in autumn rainfall in south‐eastern Australia, which has been linked to reduced runoff and moisture availability (Wasko et al. 2024). Species in the *P. bibronii* complex are autumn breeders, with slow‐developing larvae dependent on stable, shallow pools (Anstis 2017). Notably, mid‐elevation areas in our study, which had the lowest detection rates, lie within a known rain‐shadow zone (Dey et al. 2019) and may represent a hydrologically sensitive transition between coastal and sub‐alpine systems. In these sites, relatively small shifts in rainfall and evaporation could shorten breeding‐pool hydroperiods and increase the risk of recruitment failure during drought (Walls et al. 2013; Cartwright et al. 2022). Other research has also documented drought‐associated amphibian declines in parts of the study region (Scheele et al. 2012; Evans et al. 2020; Driscoll et al. 2026), and we recommend further research focus on the hydrology of breeding sites and how this factor shapes population persistence through time.

Fire and anthropogenic disturbance are major threats to some amphibians (Rowley et al. 2020; Beranek et al. 2023; Heard et al. 2023). Given the severity of the 2019–2020 ‘Black Summer’ fires in south‐eastern Australia, and their negative impacts on a broad range of fauna (Legge et al. 2022), we predicted a negative relationship between fire severity and frog occupancy. However, there was no significant relationship. Working in coastal woodlands and heathlands, Westgate et al. (2018) reported context‐dependent fire effects on *P. bibronii* occurrence, with occurrence increasing with time since fire at sites characterized by low breeding‐site density and relatively frequent burning. It is plausible that frogs in the *P. bibronii* complex show muted responses to fire more generally, if early successional conditions are favorable for adult or larval survival, and/or if fire reduces the impacts of Bd (e.g., due to negative effects of shading on temperature regimes; see Heard et al. 2015; Roznik et al. 2015; Rumschlag and Boone 2020). Nevertheless, fire severity in our study was derived from a remotely sensed raster layer and did not necessarily capture fine‐scale impacts of fire at each burnt site. As such, field‐based assessments will be important to clarify how *P. bibronii* and *P. dendyi* respond to fire regimes.

### 4.3 The Risk of Overlooking Gradual Declines

Research on amphibian declines has tended to focus on acute, rapid declines (Fisher and Garner 2020; Luedtke et al. 2023; Crawford‐Ash, Erens, et al. 2025; Crawford‐Ash, Evans, et al. 2025), with gradual declines potentially going unrecognized, especially for species that remain common in parts of their range. Without historical baselines or long‐term monitoring, these declines may be missed (Oliver et al. 2021). Cryptic amphibian species and those in remote montane or tropical regions are underrepresented in monitoring efforts (da Silva et al. 2020; Angulo et al. 2024), and targeted surveys, such as those undertaken in this study, can help close these gaps and improve detection of declines in under‐monitored species.

Well‐designed survey protocols that maximize detection are an important characteristic of robust monitoring (Scheele, Legge, et al. 2019). Our results showed detection probability peaked after nightfall, and while rainfall or moisture variables were not significant predictors of occupancy or counts, next‐day rainfall had a weak negative effect and was retained in the top count model, suggesting possible behavioral shifts before rainfall events. The weak negative relationship between probability of detecting calling males and next‐day rainfall (Table S5) indicates that surveys immediately before rain may underestimate true occupancy or counts to some degree. Additionally, surveys should span the full environmental and geographic range of a species. Although our study covered most of the range of *P. dendyi* and a portion of the southern range of *P. bibronii* , further work is needed to assess whether the scale and pattern of declines documented here are replicated elsewhere across the range of this complex.

While the *P. bibronii* complex remains widespread and relatively common at low elevations, declines and population losses have occurred in mid and higher elevation regions. These patterns likely reflect multiple interacting drivers that vary across the elevational gradient, underscoring the need to consider elevation‐specific mechanisms when assessing decline and extirpation risk. Targeted, range‐wide surveys are therefore needed to detect localized declines before they become irreversible. Recovery in mid and higher elevation sites will depend on a better understanding of the key threatening processes therein. We recommend focusing on the potential role of Bd in the declines that we document, including reservoir species dynamics, as well as quantifying the effects of fire severity and breeding site hydrology to examine potential species responses to drought and changing climatic conditions. Studies of this nature will be vital for identifying management interventions that can stem further population losses and recover depressed populations, and either facilitate natural recolonization of locally extinct populations or support reintroduction efforts.

## Author Contributions

**Jordann Crawford‐Ash:** conceptualization (equal), data curation (lead), formal analysis (equal), funding acquisition (lead), investigation (lead), methodology (equal), project administration (lead), visualization (lead), writing – original draft (lead), writing – review and editing (lead). **Stephanie Pulsford:** conceptualization (equal), investigation (supporting), methodology (equal), supervision (equal), writing – review and editing (equal). **Will Osborne:** conceptualization (equal), investigation (supporting), methodology (equal), writing – review and editing (equal). **Danswell Starrs:** conceptualization (equal), investigation (supporting), writing – review and editing (equal). **Geoffrey W. Heard:** data curation (equal), formal analysis (equal), visualization (equal), writing – review and editing (equal). **Ben C. Scheele:** conceptualization (equal), methodology (equal), supervision (equal), writing – review and editing (equal).

## Conflicts of Interest

The authors declare no conflicts of interest.

## Supporting information


**Table S1:** Site‐level occupancy rates of *Pseudophryne bibronii* complex across elevation bands. A site was considered occupied if *P. bibronii* complex was observed in any of the six surveys.
**Table S2:** Number of survey sites in each count category of *Pseudophryne bibronii* complex across three elevation bands. Count categories: 0 = no individuals detected; 1–5 individuals; 6–20 individuals; 21–50 individuals; 51–100 individuals.
**Table S3:**. Candidate single‐season occupancy models for the *Pseudophryne bibronii* complex, ranked by Akaike's Information Criterion corrected for small samples (AICc). Models with ΔAICc < 2 were model‐averaged. *K* = number of estimable parameters; ΔAICc = difference from the top model; Weight (*wᵢ*) = Akaike weight (relative support).
**Table S4:** Model‐averaged parameter estimates for the effects of site‐level covariates on occupancy probability (*ψ*) for the *Pseudophryne bibronii* complex. Boldface denotes statistical significance (*p* value < 0.05). Elevation, *Crinia signifera* maximum category and Fire Severity are standardized (z‐transformed).
**Table S5:** Model‐averaged parameter estimates for the effects of survey‐level covariates on detection probability (*p*) of calling males in the *Pseudophryne bibronii* complex. Boldface denotes statistical significance (*p* < 0.05). All variables except cosHour and sinHour are standardized (z‐transformed).
**Table S6:** Candidate cumulative link mixed models (CLMMs) for counts of the *Pseudophryne bibronii* complex, ranked by Akaike's Information Criterion corrected for small samples (AICc). Models with ΔAICc < 2 were model‐averaged. “+” denotes additive terms and “×” two‐way interactions. *K* = number of estimable parameters; ΔAICc = difference from the top model; Weight (*wᵢ*) = Akaike weight (relative support).
**Table S7:** Model‐averaged parameter estimates from cumulative link mixed models (CLMMs) for counts of the *Pseudophryne bibronii* complex. Boldface denotes statistical significance (*p* < 0.05). Elevation, next‐day precipitation and fire severity are standardized to have a zero mean and unit variance.
**Table S8:** Association between elevation (m) and *Crinia signifera* maximum count category (ordinal 0–4) across 70 sites. Spearman's *ρ* (primary) is reported with 95% percentile bootstrap CIs (10,000 resamples).
**Figure S1:** Relationship between elevation (m) and *Crinia signifera* maximum count category (0–4) across 70 sites. Points show site‐level observations; the blue line is a least‐squares fit with 95% CI (gray band).

## Data Availability

Data are available from the Zenodo Archive: https://zenodo.org/records/18883567
