# Supplementary material for: Cryptic Declines in a Widespread Australian Frog Complex
Source: Ecol Evol. 2026 Apr 14;16(4):e73384. doi: 10.1002/ece3.73384 (PMC13077290; doi:10.1002/ece3.73384)
Supplement: Supplementary file 1 — Table S1: Site‐level occupancy rates of Pseudophryne bibronii complex across elevation bands. A site was considered occupied if P. bibronii complex was observed in any of the six surveys. Table S2: Number of survey sites in each count category of Pseudophryne bibronii complex across three elevation bands. Count categories: 0 = no individuals detected; 1–5 individuals; 6–20 individuals; 21–50 individuals; 51–100 individuals. Table S3:. Candidate single‐season occupancy models for the Pseudophryne bibronii complex, ranked by Akaike's Information Criterion corrected for small samples (AICc). Models with ΔAICc < 2 were model‐averaged. K = number of estimable parameters; ΔAICc = difference from the top model; Weight (wᵢ) = Akaike weight (relative support). Table S4: Model‐averaged parameter estimates for the effects of site‐level covariates on occupancy probability (ψ) for the Pseudophryne bibronii complex. Boldface denotes statistical significance (p value < 0.05). Elevation, Crinia signifera maximum category and Fire Severity are standardized (z‐transformed). Table S5: Model‐averaged parameter estimates for the effects of survey‐level covariates on detection probability (p) of calling males in the Pseudophryne bibronii complex. Boldface denotes statistical significance (p < 0.05). All variables except cosHour and sinHour are standardized (z‐transformed). Table S6: Candidate cumulative link mixed models (CLMMs) for counts of the Pseudophryne bibronii complex, ranked by Akaike's Information Criterion corrected for small samples (AICc). Models with ΔAICc < 2 were model‐averaged. “+” denotes additive terms and “×” two‐way interactions. K = number of estimable parameters; ΔAICc = difference from the top model; Weight (wᵢ) = Akaike weight (relative support). Table S7: Model‐averaged parameter estimates from cumulative link mixed models (CLMMs) for counts of the Pseudophryne bibronii complex. Boldface denotes statistical significance (p < 0.05). Elevation, next‐day precipitat [file ECE3-16-e73384-s001.docx]

**SUPPLEMENTARY MATERIAL**

**Table S1.** Site-level occupancy rates of Pseudophryne bibronii complex across elevation bands. A site was considered occupied if P. bibronii complex was observed in any of the six surveys.

| **Elevation group** | **Total sites** | **Occupied sites** | **Occupancy rate (%)** |
| --- | --- | --- | --- |
| Low (0-500 m asl) | 14 | 10 | 71.4 |
| Mid (501-999 m asl) | 10 | 1 | 10 |
| High (1000- 1700 m asl) | 46 | 13 | 28.3 |

**Table S2.** Number of survey sites in each count category of Pseudophryne bibronii complex across three elevation bands. Count categories: 0 = no individuals detected; 1–5 individuals; 6–20 individuals; 21–50 individuals; 51–100 individuals.

|  | **Count of individuals** | | | | |
| --- | --- | --- | --- | --- | --- |
| **Elevation band** | **0** | **1-5** | **6-20** | **21-50** | **51-100** |
| High (1000-1700 m) | 33 | 8 | 4 | 1 | 0 |
| Mid (501- 999 m) | 9 | 1 | 0 | 0 | 0 |
| Low (0-500 m) | 4 | 1 | 4 | 4 | 1 |

**Table S3**. Candidate single-season occupancy models for the Pseudophryne bibronii complex, ranked by Akaike’s Information Criterion **corrected for small samples (AICc).** Models with **ΔAICc < 2** were model-averaged. **K =** number of estimable parameters**; ΔAICc** = difference from the top model; **Weight (wᵢ) =** Akaike weight (relative support).

| **Model** | **K** | **AICc** | **ΔAICc** | **Weight (wᵢ)** |
| --- | --- | --- | --- | --- |
| Elevation + *Crinia signifera* count | 13 | 251.60 | 0.00 | 0.331 |
| Elevation + *Crinia signifera* count + Fire severity | 14 | 252.70 | 1.10 | 0.192 |
| Elevation + Fire severity | 13 | 254.40 | 2.74 | 0.084 |
| *Crinia signifera* count | 12 | 254.70 | 3.07 | 0.071 |
| Elevation + *Crinia signifera* count + Canopy cover | 14 | 254.80 | 3.12 | 0.070 |
| *Crinia signifera* count + Fire severity | 13 | 255.30 | 3.71 | 0.052 |
| Elevation | 12 | 255.50 | 3.84 | 0.049 |
| Elevation + *Crinia signifera* count + Fire severity + Canopy cover | 15 | 255.70 | 4.09 | 0.043 |
| *Crinia signifera* count + Fire severity + Canopy cover | 14 | 256.50 | 4.86 | 0.029 |
| *Crinia signifera* count + Canopy cover | 13 | 256.70 | 5.04 | 0.027 |
| Elevation + Fire severity + Canopy cover | 14 | 257.50 | 5.88 | 0.018 |
| Fire severity | 12 | 257.70 | 6.09 | 0.016 |
| Elevation + Canopy cover | 13 | 258.20 | 6.54 | 0.013 |
| Fire severity + Canopy cover | 13 | 259.80 | 8.12 | 0.006 |
| Canopy cover | 12 | 263.00 | 11.33 | 0.001 |

**Table S4.** Model-averaged parameter estimates for the effects of site-level covariates on occupancy probability (ψ) for the Pseudophryne bibronii complex. Boldface denotes statistical significance (p value < 0.05). Elevation, Crinia signifera **maximum category and Fire Severity are standardised (z-transformed).**

| **Parameter** | **Estimate** | **SE** | **z value** | **p value** | **95% CI (lower- upper)** |
| --- | --- | --- | --- | --- | --- |
| **Intercept** | **-0.706** | **0.297** | **2.377** | **0.017** | -1.288 − 0.124 |
| **Elevation** | **-1.417** | **0.619** | **2.290** | **0.022** | -2.629 − 0.204 |
| Crinia signifera **maximum category** | **1.469** | **0.639** | **2.299** | **0.022** | 0.217 − 2.721 |
| Fire severity | 0.307 | 0.540 | 0.568 | 0.570 | 0.325 − 2.001 |

**Table S5.** Model-averaged parameter estimates for the effects of survey-level covariates on detection probability (*p*) of calling males in the Pseudophryne bibronii complex. Boldface denotes statistical significance (P < 0.05). All variables except cosHour and sinHour are standardised (z-transformed).

| **Parameter** | **Estimate** | **SE** | **z value** | **p value** | **95% CI (lower- upper)** |
| --- | --- | --- | --- | --- | --- |
| Intercept | 1.860 | 0.735 | 2.533 | **0.011** | 0.421 − 3.309 |
| Daily temperature | -0.377 | 0.417 | 0.905 | 0.365 | -1.194 − 0.440 |
| Next-day precipitation | -0.690 | 0.453 | 1.522 | 0.128 | -1.578 − 0.199 |
| Wind | -0.428 | 0.478 | 0.895 | 0.371 | -1.365 − 0.510 |
| Humidity | -0.141 | 0.449 | 0.313 | 0.756 | -1.021− 0.740 |
| Soil moisture | 0.487 | 0.424 | 1.148 | 0.251 | -0.344 − 1.319 |
| Cloud cover | 0.134 | 0.448 | 0.299 | 0.765 | -0.744 − 1.013 |
| cosHour | 2.023 | 0.777 | 2.604 | **0.009** | 0.501 − 3.545 |
| sinHour | 0.691 | 0.530 | 1.303 | 0.193 | -0.349 − 1.730 |
| Habitat disturbance | 0.159 | 0.596 | 0.267 | 0.790 | -1.009 − 1.326 |

**Table S6.** Candidate cumulative link mixed models (CLMMs) for counts of the *Pseudophryne bibronii* complex, ranked by Akaike’s Information Criterion **corrected for small samples (AICc).** Models with **ΔAICc < 2** were model-averaged. “+” denotes additive terms and “×” two-way interactions. **K =** number of estimable parameters**; ΔAICc** = difference from the top model; **Weight (wᵢ) =** Akaike weight (relative support).

| **Model** | **K** | **AICc** | **ΔAICc** | **Weight** |
| --- | --- | --- | --- | --- |
| Elevation + Night (vs. Day) + Next-day precipitation | 7 | 163.034 | 0.000 | 0.290 |
| Elevation + Night (vs. Day) + Next-day precipitation + Fire severity | 8 | 163.626 | 0.593 | 0.216 |
| Elevation + Night (vs. Day) | 6 | 165.176 | 2.142 | 0.099 |
| Elevation × *Crinia signifera* count + Night (vs. Day) + Next-day precipitation | 9 | 165.675 | 2.641 | 0.077 |
| Elevation + Night (vs. Day) + Fire severity | 7 | 165.884 | 2.850 | 0.070 |
| Elevation + Next-day precipitation | 6 | 166.046 | 3.013 | 0.064 |
| Elevation × *Crinia signifera* count + Night (vs. Day) + Next-day precipitation + Fire severity | 10 | 166.467 | 3.434 | 0.052 |
| Elevation + Next-day precipitation + Fire severity | 7 | 167.761 | 4.728 | 0.027 |
| Elevation × *Crinia signifera* count + Night (vs. Day) | 8 | 168.073 | 5.039 | 0.023 |
| Elevation × *Crinia signifera* count + Next-day precipitation | 8 | 168.134 | 5.100 | 0.023 |
| Elevation only | 5 | 168.675 | 5.641 | 0.017 |
| Elevation × *Crinia signifera* count + Night (vs. Day) + Fire severity | 9 | 168.718 | 5.684 | 0.017 |
| Elevation × *Crinia signifera* count + Next-day precipitation + Fire severity | 9 | 169.926 | 6.892 | 0.009 |
| Elevation + Fire severity | 6 | 170.483 | 7.449 | 0.007 |
| Elevation × *Crinia signifera* count | 7 | 170.905 | 7.872 | 0.006 |
| Elevation × *Crinia signifera* count + Fire severity | 8 | 172.602 | 9.568 | 0.002 |

**Table S7.** Model-averaged parameter estimates from cumulative link mixed models (CLMMs) for counts of the Pseudophryne bibronii complex. Boldface denotes statistical significance (*P* < 0.05). Elevation, next-day precipitation and fire severity are standardised to have a zero mean and unit variance.

| **Predictor** | **Estimate** | **SE** | **z value** | **p value** |
| --- | --- | --- | --- | --- |
| Elevation | **-1.517** | **0.577** | **2.630** | **0.009** |
| Night (vs Day) | **1.334** | **0.578** | **2.310** | **0.021** |
| Next-day precipitation | -2.775 | 1.781 | 1.558 | 0.119 |
| Fire severity | -0.909 | 0.687 | 1.324 | 0.186 |

**Table S8.** Association between elevation (m) and Crinia signifera maximum count category (ordinal 0–4) across 70 sites. Spearman’s ρ (primary) is reported with 95% percentile bootstrap CIs (10,000 resamples).

| **Correlation** | **Estimate** | **95% CI** | **Test statistic** | **p** | **n** |
| --- | --- | --- | --- | --- | --- |
| Spearman ρ | -0.12 | -0.35 − 0.12 | S = 63,961 | 0.326 | 70 |

**
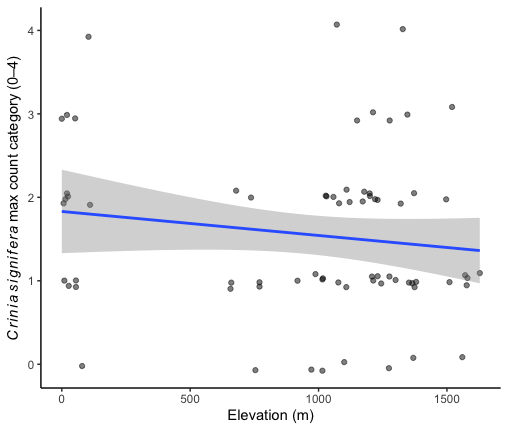
**

**Figure S1.** Relationship between elevation (m) and Crinia signifera maximum count category (0–4) across 70 sites. Points show site‐level observations; the blue line is a least-squares fit with 95% CI (grey band).
